# Diagnostic Aspects of Veterinary and Human Aspergillosis

**DOI:** 10.3389/fmicb.2018.01303

**Published:** 2018-06-21

**Authors:** Daniel Elad, Esther Segal

**Affiliations:** ^1^Department of Clinical Bacteriology and Mycology, Kimron Veterinary Institute, Bet Dagan, Israel; ^2^Department of Clinical Microbiology and Immunology, Sackler Faculty of Medicine, Tel Aviv University, Tel Aviv, Israel

**Keywords:** aspergillosis, animal, human, diagnosis, antimycotic drugs

## Abstract

The genus *Aspergillus* is composed of more than 300 species, a fraction of which are involved in animal or human infections mostly following environmental exposure. Various risk factors (i.e., immunosuppression, tuberculosis) have been recognized for human whereas for veterinary infections, unhygienic management, trauma, anatomical conformation of the skull, or suspected immunological deficiencies have been suggested. In animals, aspergillosis is mostly sporadic but in some circumstance such as infections on poultry farms may involve the whole flock. Since the high prevalence of immunosuppression in human patients has not been mirrored in veterinary medicine, and although to the best of our knowledge, no comprehensive data on the prevalence of aspergillosis in animals has been published, their epidemiology has not changed during the last decades. The impact of these infections may be economic or if they are incurable, sentimental. The objective of the first part is to describe the diagnosis of the main clinical entities caused by *Aspergillus* spp. in animals. It includes disseminated canine aspergillosis, canine and feline sino-nasal and sino-orbital aspergillosis, guttural pouch mycosis in horses, mycotic abortion in cattle, mycotic keratitis in horses, and avian aspergillosis. When pathogenesis and clinical aspects are relevant for diagnosis—they will be addressed as well. The second part deals with human aspergillosis, which is a multifaceted disease, manifested in a spectrum of clinical entities affecting one or more organs. Diagnosis is based on the clinical manifestation, supported and confirmed by laboratory means, involving the classical approach of demonstrating the etiological agent in the clinical specimens and in culture. Noncultural methods, such as antigen detection and/or molecular assays to detect fungal nucleic acids or protein profiles, are used as well. The isolation and identification of the fungus allows the determination of its susceptibility to antifungal drugs. Thus, antifungal susceptibility testing maybe considered as part of the diagnostic process, which is of relevance for management of the infection. In this review article, the part dealing with diagnostic aspects of aspergillosis in humans concentrates on susceptibility testing of *Aspergillus* spp. to antifungal drugs and drug combinations. The technologies and methods of susceptibility testing are described and evaluated.

## Veterinary Infections Caused by *Aspergillus* Spp.

### Introduction

Veterinary mycology differs from human mycology mainly in two important aspects. Since the 1980s the phenomenon of immunosuppression, pathological or iatrogenic, has affected an ever-increasing number of people. This has led to the emergence of fungal infections many of which were caused by opportunistic fungi, previously not or rarely involved in human mycoses (Enoch et al., 2017). A byproduct of this change in fungal infection epidemiology was the discovery of a large variety of antimycotic drugs, many of which are prohibitively expensive for veterinary use. This phenomenon has bypassed animals since the gamut of fungi involved in veterinary mycoses has remained largely unchanged, with differences stemming primarily from factors such as improved diagnostic methods and raised awareness (Elad, 2011). Susceptibility testing of animal *Aspergillus* isolates is exceptional due to their limited use and their low predictive value stemming from the animals’ different anatomical and physiological characteristics.

The second aspect that differentiates veterinary from human mycology is the economic one. The price has a significantly higher impact on decisions to diagnose, identify the etiology, and treat disseminated fungal infections in animals, especially livestock. Thus, the economic or sentimental value of the animal/s dictates the actions (such as environmental decontamination, antimycotic therapy, or euthanasia) to be taken in case of animal aspergillosis.

An accurate diagnosis of animal fungal infections in general and aspergillosis in particular is of significant importance to determine the prognosis, remedial steps to be taken, and the choice of therapy, especially considering the differences in susceptibility of the various species of the fungus. Moreover, the importance of the etiology’s identification stems from the fact that some *Aspergillus* infections cannot be differentiated clinically from infections caused by other pathogens that require a different therapeutic approach and/or pose a zoonotic threat (see mycotic abortion and brucellosis below). The primary identification of the fungus may be based on morphology (this may necessitate the use of specific media such as Czapek-Dox agar). Due to differences in the prognosis and susceptibility of morphologically undistinguishable fungi, the exact identification of the etiology is of importance. It may require the sequencing of the Internal Transcribed Spacer gene and often additional genes such as those of tubulin and/or calmodulin (Barrs et al., 2014). Serological methods were tested in animals in a limited number of cases and do not allow obtaining unequivocal conclusions as to the mycoses’ nature (Schultz et al., 2008).

Most cases of animal aspergillosis are sporadic. Immunosuppression, either pathologic or iatrogenic, that has been the main predisposing factor to human mycoses in general and aspergillosis in particular, is much rarer in animals, and thus is not considered as a significant risk factor. Infection source may vary but is mostly environmental. Heavy fungal loads may be the source of massive poultry infections whereas trauma is usually assumed to cause ophthalmic infections. Disseminated or rhino-nasal/rhino-orbital aspergillosis seem to be associated with breed predisposition related to immune deficiencies the former and skull conformation the latter. The detailed basis of this predisposition is still to be elucidated.

Human activities that may promote aspergillosis include the use of contaminated intramammary tubes (Elad et al., 1995) or unhygienic management, especially of poultry farms, but in most cases such activities have no bearing on other infections.

Several well-defined clinical entities have been described and are reviewed.

### Disseminated Canine Aspergillosis

Disseminated canine aspergillosis (DCA) affects primarily German Shepherd breed dogs, with females being over-represented (Taylor et al., 2015). It is caused most frequently by *Aspergillus terreus* or, more rarely by two other species belonging to the *Terrei* group, namely *A. carneus* and *A. alabamensis*. Other *Aspergillus* spp. that may cause disseminated canine mycoses include *A. deflectus, A. fumigatus, A. niger, A. flavus, A. flavipes, A. versicolor*, or unspecified *Aspergillus* spp. Clinical signs are non-specific and include lethargy, weight loss, central nervous system signs, and ataxia due to musculoskeletal lesions (Schultz et al., 2008). Abnormal clinical pathological test results may vary and result from the inflammatory process and/or dysfunctions of the affected organs. Since these signs are non-specific, diagnosis of DCA is often made after prolonged periods of therapeutic attempts aimed at other pathogens, with advanced dissemination by the time the correct etiology is identified (Zhang et al., 2012).

*In vivo* diagnosis may be based on the isolation of the fungus from biopsies and/or urine (Bruchim et al., 2006) reflecting the dissemination of the fungus to the kidneys. In urine sampled by cystocentesis to avoid contamination, the clusters of hyphae seen microscopically are a clear indication of a disseminated fungal infection, albeit not necessarily caused by *Aspergillus* spp. Thus if positive, this test that can easily perform animal-side provides a quick diagnosis of the infection’s nature. For some *Aspergillus* spp., especially *A. terreus*, the presence of accessory conidia (aleurioconidia) in tissue and/or culture is an important indication as to the identity of the infection’s etiology (Elad et al., 2008). In addition, a commercial kit (Platelia Aspergillus, BioRad, United States), aimed at the detection of *Aspergillus* galactomannan in serum or bronchoalveolar lavage, by an immunoenzymatic sandwich microplate assay has been assessed by Garcia et al. (2012) in dogs. They found that dogs with DCA had significantly higher values than suspected or control animals. Sensitivity was 92 and 88% for serum and urine samples, respectively, whereas specificity for the same samples was 86 and 92%. Increasing the cutoff value for human samples from 0.5 to 1.5 raised the specificity of the serum and urine samples to 93% without impacting the sensitivity. Corrigan et al. (2016) reported that when serial samples from two dogs under antimycotic therapy were examined with the kit, the values decrease for one and remained high for the other. The latter showed some clinical improvement but relapsed whereas the former survived. Conclusive diagnosis of CDA should, however, not be based exclusively on serodiagnosis (Bentley et al., 2018).

Taylor et al. (2015) examined seven dogs with CNS aspergillosis by magnetic resonance imaging (MRI). No aberrant findings were present in the MRI examination of the central nervous system of three dogs and those found in three others were variable (the 7th dog had discospondylitis). Moreover, they note that, unlike human CNS aspergillosis, in which typical changes can be observed by MRI, similar infections in dogs cannot be diagnosed solely by MRI and the latter should be complemented by other test in cases of suspected aspergillosis.

Post mortem diagnosis is based on the presence of hyphae in the organ lesions, their isolation, and identification. It should be stressed that other molds cannot be differentiated from *Aspergillus* spp. at the histopathological examination unless immunohistochemistry is applied (Pérez et al., 1996). Moreover, to accurately identify the mold species involved, such as those belonging to the section *Terrei*, sequencing the ITS gene alone may be insufficient and additional genes such as that encoding for tubulin should be sequenced (Burrough et al., 2012).

Due to the normally late presentation of the dogs and the poor record of antimycotic therapy success, animals are only rarely treated (Zhang et al., 2012). Recently, treatments with novel drugs, including voriconazole, posaconazole, and echinocandin have been attempted (Schultz et al., 2008). These drugs are currently too expensive for veterinary use, especially considering the fact that treatment has to be prolonged, possibly for the animal’s whole life. Even if seemingly successful, animals have relapsed after the treatment’s cessation, sometimes after years. Only three dogs have been reported to survive three or more years after therapy cessation (Kelly et al., 1995; Watt et al., 1995; Corrigan et al., 2016). Nevertheless, further therapeutic protocols including antimycotic drug combinations, dosages, and treatment periods should be tested to find a suitable treatment for DCA. Currently this is possible, due to economic considerations, only in an experimental setup and thus in a limited number of cases.

### Sino-Nasal and Sino-Orbital Aspergillosis

Sino-nasal aspergillosis (SNA) afflicts more frequently dolichocephalic and mesocephalic dogs (Peeters and Clercx, 2007). They are caused primarily by *A. fumigatus*, although other molds are occasionally isolated (Talbot et al., 2014). Animals are regularly exposed to the fungal spores that are common in the environment. The mucociliary apparatus and the innate immune reaction of the lungs are usually sufficient to prevent the development of the infection and if they fail, SNA may develop (Sharman and Mansfield, 2012). In addition to host risk factors, fungal virulence factors such as the small size of conidia allowing them easy propagations in the host and metabolites such *A. fumigatus* gliotoxin that inhibits the mucociliary clearing capabilities may play a significant role in allowing the fungus to colonize the mucosae and lead to the establishment of a fungal mat (Peeters and Clercx, 2007). Deeper tissues are usually not invaded but occasionally the cribriform plate may be affected, most probably by fungal metabolites and the inflammatory reaction, resulting in the opening of a passageway to the central nervous system (Sharman and Mansfield, 2012). This process may have therapeutic implications (see below).

Initial clinical signs, sometimes present for prolonged periods (months, years) are non-specific and include those characterizing upper respiratory tract infections (URTIs): mucopurulent nasal discharges that may become hemorrhagic with eventual depigmentation of the nasal plane (Sharp et al., 1991). A differential diagnosis has to be made with other clinical entities with similar symptoms such as malignancies or foreign bodies (Cohn, 2014).

For the diagnosis of SNA various techniques may be employed, usually requiring the combination of more than one method to be precise.

Diagnostic methods involving imaging procedures include radioscopy, computerized tomography (CT), and MRI. The latter two, although more expensive and less available, are superior to the former, each having advantages and disadvantages in revealing the variety of possible lesions associated with SNA (Cohn, 2014). Karnik et al. (2009), however, report that CT was not always able to discriminate between SNA and neoplasia. These techniques do not discriminate between cases of SNA caused by *Aspergillus* spp. and other fungi. *Aspergillus* spp., however, cause most of these infections (Talbot et al., 2014) and thus may be empirically considered as the etiology until proven otherwise.

Among non-cultural methods, cytology of nasal discharge or blind swabs has low sensitivity and specificity as finding fungal elements may not be linked unequivocally to infection since their presence may be the result of other factors such as environmental contamination. Examining brush smears or squash biopsies significantly increased the sensitivity of cytology (De Lorenzi et al., 2006) especially if incubated at 37°C (Sharman and Mansfield, 2012). Comparably to the imaging techniques mentioned above, fungal hyphae found in cytological samples cannot unequivocally be identified as *Aspergillus* spp. since they are indiscernible from several other mold species (albeit with much lower prevalence), possibly having different prognostic and therapeutic significance. The sensitivity and specificity of serological tests are not uniform and quantitative DNA assays do not discriminate between infected and uninfected dogs as well as dogs suffering from some other nasal afflictions (Sharman and Mansfield, 2012).

Various approaches for SNA therapy, local and systemic, mostly based on azoles, have been reported (Peeters and Clercx, 2007). When administered alone, the therapeutic value of systemic drugs is limited, possibly due to the non-invasive nature of the mycosis. Topical treatment may be administered through catheters or by trephination of the frontal sinuses. They allow direct contact between the drugs and the fungal mat and thus are considered to be more efficacious. Additional treatment techniques have been described. Topical therapy is contraindicated in cases of cribriform plate involvement since the drug may leak into the cranium (Peeters and Clercx, 2007). One feline case, caused by *A. fumigatus*, was treated successfully, after debridement, with posaconazole, following a relapse after itraconazole therapy (Tamborini et al., 2016).

Sino-orbital aspergillosis (SOA) afflicts mostly cats and may be a complication of SNA. Brachycephalic skull conformation may increase the risk (Barrs et al., 2012). While SNA is mostly caused by *A. fumigatus*, the development of SOA is associated with a recently described species, *Aspergillus felis* (*Neosartorya*-morph). *A. felis* is a heterothallic mold, able to grow at 45°C but not 50°C, differentiating it from the morphologically similar *A. udagawae* and *A. fumigatus*, respectively (Barrs et al., 2013). *A. felis* is more invasive than *A. fumigatus* and it may cause lysis of the orbital lamina and pass into the orbital cavity, resulting in the development of a granuloma and exophthalmos (Hamilton et al., 2000). Subsequently other adjacent organs may be invaded (Barrs et al., 2013). Moreover, *A. felis* is more resistant to azoles than *A. fumigatus*, and thus the prognosis is poorer (Barrs et al., 2007). This emphasizes the clinical importance of the exact taxonomic identification of the infection’s etiology, preferably by molecular methods. In dogs, although the lysis of the orbital lamina may be present in SNA caused by *A. fumigatus*, the development of SOA aspergillosis is rare (Barrs et al., 2014).

Whitney et al. (2013) assessed the possibility of sero-diagnosis of SOA by a commercial ELISA kit (Platelia Aspergillus, BioRad, United States) that detects galactomannan. They examined cats with proven URTIs caused by (URTIs) aspergillosis (confirmed by cultural and molecular methods), cats with URTIs not caused by *Aspergillus* spp., cats without URTIs but treated with β-lactam antibiotics, and healthy cats. Only 3 cats out of 13 of the *Aspergillus* spp. URTI had positive results as did almost one-third of the non-*Aspergillus* spp. infected groups. Consequently, this method was found by the authors as having low sensitivity and limited specificity.

Barrs et al. (2015) evaluated the sensitivity, specificity and positive and negative predictive value of indirect ELISA and agar gel immunodiffusion (AGID) to diagnose SNA or SOA. They compare sera of cats affected by SNA or SOA caused by *A. fumigatus* or several cryptic species including *A. felis*, to those of cats with non-fungal upper respiratory infections and healthy cats. The sensitivities were 43 and 95.2% and the specificities were 100% and 92–92.9% for the AGID and ELISA test, respectively. No differences between the tested species were found. Assessing IgA titers did not improve the results obtained by IgG alone (Taylor et al., 2016).

The difficulty and complexity of SOA treatment are epitomized in a series of SNA or SOA cases in cats (Barrs et al., 2012). Various treatment protocols including amphotericin B, itraconazole, posaconazole, and terbinafine alone or in combination have been reported. Other methods include out of seven SOA cases, one cat responded to amphotericin B and itraconazole, relapsed after 8 months, then was treated apparently successfully with posaconazole and terbinafine, relapsed after 19 months and did not improve with liposomal amphotericin B and posaconazole. Finally, treatment with caspofungin followed by posaconazole resulted in remission for the 12 months, when the article was written. It has to be noted, however, that some of these drugs such as liposomal amphotericin B and caspofungin are currently too expensive to be used in veterinary medicine except under experimental conditions.

### Guttural Pouch Mycosis

The guttural pouch (GP) is a diverticulum of the Eustachian tubes in one toed ungulates (*Peryssodactila*), hyraxes, some bats, and the American forest mouse. Among these, horses are the only domestic animals that have this organ (Nation, 1978). The GPs are in contact with some major arteries and nerves which may be affected when the walls of the GP are eroded by the fungi (Davis and Legendre, 1994). This may result in potentially fatal epistaxis (while the animal is at rest) and/or paralysis of various cranial nerves leading, among others, to dysphagia (Freeman, 2015). Microorganisms, including fungi, have been found in healthy GPs (Manglai et al., 1999) and thus predisposing factors are necessary to allow the evolution of an infection. Although several hypotheses were suggested as to the nature of such factors, they thus far have remained unidentified (McLaughlin and O’brien, 1986). Fungi involved are primarily *A. fumigatus* and *A.* (*Emericella*) *nidulans* but other *Aspergillus* spp. have been reported (Freeman, 2015).

Presentation and initial diagnosis are based on the clinical signs (epistaxis, paralysis). Thus, in most cases the infection is already in an advanced stage (Lepage et al., 2004). In the subsequent endoscopy areas of necrotic tissue with fungal mats may be seen (Dobesova et al., 2012). The definitive diagnosis is based on the histopathological demonstration of hyphal invasion of tissue and the fungus’ isolation. It is noteworthy that, similar to what has been described in canine SNA, some samples do not yield fungal growth in culture (Seyedmousavi et al., 2015).

Guillot et al. (1997) compared three serological tests to diagnose equine aspergillosis. They examined 12 horses with endoscopically verified fungal plaques in the GP and 12 healthy controls by counter immunoblot and ELISA. *Aspergillus* spp. was isolated from only two horses (the remaining were either culture negative or not cultured). Antibodies were found by the ELISA test in both infected and healthy horse, possibly indicating previous exposure to the fungus. In the immunoblot tests, two antigens (22 and 26 kd) were detected in all the horse with GP mycosis. While the test for the 22 kd antigen resulted positive in two control samples, the one for 26 kd was negative in all the samples of this group.

Therapy is mostly surgical and aimed at preventing or treating epistaxis (Freeman, 2015). This may be accompanied by antimycotic treatments by various drugs (Greet, 1987). Surgical removal of fungal plaques followed by antimycotic treatment prevented their reformation (Church et al., 1986). In cases of dysphagia, antmycotic therapy is the only alternative but the chances of recovery are poor (Freeman, 2015) since this approach can solve the fungal infection but it has no impact on the paralysis poor (Church et al., 1986). One case was, however, reported in which the fungal infection was successfully treated with a combination of itraconazole and topical enilconazole (Davis and Legendre, 1994) and subsequently the dysphagia regressed within a few weeks.

### Mycotic Abortion

Mycotic abortion with *Aspergillus* spp. as etiological agent was reported in various animals such as horses (Monga and Mohapatra, 1980; Hong et al., 1993) and pigs (Todd et al., 1985). Mycotic abortions in cattle, however, have been the focus of most publications. *A. fumigatus* is the most prevalent etiology, although other species such as *A. terreus* have been reported as well (Elad and Bernstein, 1987).

The pathogenesis of mycotic abortions in ruminants has significant implication for the interpretation of the diagnostic results. The contact between the fetal and the maternal part of the ruminant placenta is special mainly in two characteristics: (a) it is not contiguous but occurs in contact organs called placentomes, composed of the fetal part—the cotyledon and the maternal part—the caruncle and (b) there is no direct contact between the fetal and the maternal blood vessels. Thus, infectious agents that spread hematogenously do not pass from the maternal to the fetal blood but infect the placentome and, if the pathological process is acute enough to lead to an early abortion, the agent will not be found in the fetal organs. This happens in about 70% of cases. If, however, the abortion is delayed, the microorganism will spread through the amniotic fluid first to the fetus’ stomach (abomasum), then to the lungs, and finally to the other organs. Since the placenta is often not found or is in a state that does not permit its microbiological examination, examining the fetus alone does not reveal the abortive agent (Sheridan et al., 1985).

Bovine mycotic abortions (BMAs) occur mostly in the third trimester of pregnancy and are mostly sporadic although they are an important cause of mycotic abortions in some countries (Hugh-Jones and Austwick, 1967). Symptoms of BMA include modifications to the placenta that becomes thick and “leathery.” Cotyledons are thickened and may have necrotic centers. This may also occur in abortions caused by *Brucella* spp. and, considering the significant zoonotic potential of this microorganism, appropriate precautions should be taken until the abortion’s etiology is ascertained. In addition, raised, hyperkeratotic plaques may be present (Glover et al., 2011) on the fetus’ skin although the frequency of this symptom is uncertain.

Laboratory diagnosis is based on culture of the fungus from the placenta and/or the fetus and its identification. Since the culture of *Aspergillus* spp. may be the result of contamination, it is imperative to demonstrate tissue invasion by histopathology (Jensen et al., 1991). The mycological identification of the fungus causing the abortion may sometimes not be possible due to contaminant overgrowth. In a comparison between three diagnostic methods, Jensen et al. (1991) found a relatively low (kappa = 0.28) relationship between the attempted histopathological and mycological identification of the fungi. A better agreement was found between immunofluorescence and histopathology or mycology (kappa = 0.4 and 0.48, respectively). Jensen et al. (1993) assessed by inhibition ELISA the presence of galactomannan in the sera and urine of calves infected experimentally intravenously with *A. fumigatus*, cows with mycotic placentitis and abortion (confirmed by histopathology and culture), cows that aborted for other reasons, cows that did not abort but had other infections and healthy cows at the slaughterhouse. The results showed that the test was neither sensitive nor specific enough to be considered a reliable means to diagnose mycotic abortions in cattle.

In addition to the clinical and microbiological diagnosis, the possibility of serological diagnosis of mycotic abortions in cattle and sheep was assessed (Corbel et al., 1973; Wiseman et al., 1984; Jensen et al., 1991, 1993), but to the best of our knowledge, these experiments did not result in diagnostic tests.

### Mycotic Keratitis

Mycotic keratitis (MK) may afflict a variety of animal species with trauma providing the portal of entry for the infecting agent (Aho et al., 1991). Horses seem to be the animals most frequently affected, possibly due to the lateral location and protrusion of their eyes (Sherman et al., 2017). In fact, this syndrome comprises about one-third of cases of equine keratitis. *Aspergillus* spp., especially *A. fumigatus*, often the etiological agent of these infections (Aho et al., 1991) has been isolated also from eyes of healthy horses (Sherman et al., 2017). Proteases produced by these fungi provide an important pathogenic mechanism (Gopinathan et al., 2001).

Gaarder et al. (1998) divided MK cases into five types. The most severe form is characterized by the development of a furrow in the cornea. Most of these cases necessitated enucleation or even exenteration. The authors suggested that blocking of deep vascularization by the furrow impeded the formation of the granulation tissue required for healing.

Medical treatment with drugs such as voriconazole, miconazole, natamycin, itraconazole, or oral fluconazole suffices to heal some cases while surgical intervention may be necessary in other cases (Sherman et al., 2017).

### Avian Aspergillosis

Avian aspergillosis afflicts domestic and wild birds, free and captive. Some species such as penguins seem to be over-represented (Samanta and Bandyopadhyay, 2017) but their increased susceptibility has not been unequivocally confirmed (Fischer and Lierz, 2015). Young birds seem to be more susceptible to acute aspergillosis (Fischer and Lierz, 2015). It may be chronic, resulting from the action of predisposing factors such as stressors, husbandry, or other pathogens or acute due to exposure to high spore concentrations. A possible connection between spore rich environments and human colonization has been suggested (Cafarchia et al., 2014). Although not a mycotic infection and thus beyond the scope of this review, it is noteworthy that *Aspergillus* spp. may contaminate hatcheries causing significant economic damages (Hamet et al., 1991). Fungi infecting birds must be able to grow at the relatively high body temperature of these animals that may surpass 40°C (Prinzinger et al., 1991). *Aspergillus* spp. in general and *A. fumigatus* in particular are the main etiological agents involved (Neumann, 2016).

A special feature of bird anatomy, the air sacs or coelomic cavities, facilitates the systemic dissemination of fungal spores. Air sacs are hollow organs, distributed throughout the body and inside bones. In pelicans they are present also in the subcutis. They communicate with the lungs and thus spores that reach the lower respiratory tract can disperse to other body compartments (König et al., 2016).

Clinically, the first signs include breathing difficulties and wheezing due to the obstruction of airways by fungal granulomata (Girma et al., 2016). Subsequently general symptoms such as lethargy, inappetence, diarrhea, and feather ruffling may appear (Pardeike et al., 2016). In other cases, wasting may be the only symptom (Neumann, 2016). Pathological changes include granulomata in the lungs and fungal plaques in the air-sacs are (Girma et al., 2016).

Since clinical symptoms are not specific for aspergillosis, additional tests are necessary to confirm the diagnosis (Neumann, 2016). In cases of flocks, husbandry deficiencies can provide the initial indication to fungal infections (Fischer and Lierz, 2015).

For individual birds, tests may include radiology, CT, or MRI scans (Schwarz et al., 2016) and endoscopy to reveal occlusions in birds with straight tracheae (if this organ is convoluted, such as in some crane species, this approach is not possible). In addition, endoscopy allows sampling of the lesions and culturing of the etiological agent. The detection of a fungal toxin, fumigaclavin, has been suggested to indicate avian aspergillosis but the conditions in which it may be detected have to be further clarified (Seyedmousavi et al., 2015). Aberrant clinical pathological test results are mostly the function of the organ involved. Other methods include protein electrophoresis on cellulose acetate or agarose gel film (for a detailed description of the method see Werner and Reavill, 1999). A decrease in albumin or serum proteins was found to be negative prognostic indicators in penguins (Werner and Reavill, 1999). These findings were confirmed by Naylor et al. (2017) who assessed the prognostic value of the plasma protein value in 183 Gentoo penguins (*Pygoscelis papua papua*), as determined by agarose gel electrophoresis. They found that the negative predictive value of an increase in the albumin/globulin ratio to be high whereas the positive predictive value was limited. It is noteworthy, however, that since standard plasma protein profiles for many bird species have not been defined, the interpretation of these tests may be problematic (Fischer and Lierz, 2015).

Biopsies may reveal fungal hyphae whereas immunohis-tochemistry may indicate whether the infecting fungus belongs to the genus. Challa et al. (2015) assessed the specificity of these tests by using polyclonal anti-*Aspergillus* rabbit antibodies on 50 paraffin-embedded samples. Forty-seven of these consisted of tissue samples in which hyphae were seen and three of cultures. *Aspergillus* spp. were cultured from 25 of the tissue samples. The remaining tissue samples and the cultures were of a variety of fungi. They found that 88% of the proven aspergillosis cases were positive and no cross reaction with the other fungi. Consequently they concluded that immunohistochemistry has the potential to be used as a supplementary test to diagnose avian aspergillosis. Moreover, they reviewed previously published studies and stressed the impact of the method employed on the results.

The therapeutic approach to avian aspergillosis differs in flocks or individual birds. In the former, husbandry improvements such as decrease in stressors, better ventilation, and uncontaminated feed and environment are required. For individual birds, various treatments have been described (Krautwald-Junghanns et al., 2015). In some cases, such as wild birds, their periodic capture, necessary for repeated administration of parenteral antimycotic drugs during long periods, may be impractical due to the resulting stress. Moreover, the fungus’ sequestration from the blood stream may result in it not being exposed to the drug (Girma et al., 2016). Consequently, oral antimycotics are preferred and various approaches such as nebulization or the use of nanosuspensions have been attempted (Wlaź et al., 2015; Pardeike et al., 2016). Since therapeutic protocols have not been validated specifically for birds in general and for the various species in particular, necessary care should be taken, especially considering the prolonged time required to complete the treatment (Tartor and Hassan, 2017). In cases in which anesthesia is necessary such as surgery for air sac exposure to alleviate respiratory distress or imaging tests, the eventual impact of air way obstructions or other lung lesions upon the anesthetic process must be considered.

### Future Prospects

Improved diagnostic methods and economically sustainable therapeutic options could significantly improve the prognosis of animal aspergillosis. Reports of adapting modifications of human kits such as that aimed at galactomannan detection to animals, especially pets, are few and should be the object of more detailed assessment resulting in clear use recommendations. In addition, results of imaging techniques have also been found to be often unreliable and should be further investigated.

While great advances in antifungal therapy have been made in the last years, the price of the newer drugs is prohibitive for animal use especially considering the necessity of extended, possibly life-long, treatments. This has limited their use to a very low number of cases, mostly for scientific studies. It is expected that the price of these drugs will decrease in the future, but the development of specific antifungal drugs for veterinary use should be considered as well.

A special case of future research should try to define the immunologic basis of canine disseminated aspergillosis. This would permit the exclusion of dogs carrying the faulty gene to be excluded from the pedigree lists (as has been done for hip dysplasia in GS dogs) and reduce the number of cases.

## Human Aspergillosis: *In Vitro* Assessment of Susceptibility to Drugs and Drug Combinations Against *Aspergillus*

### Introduction

Human aspergillosis is a multifaceted disease, including ear, sinus, eye, skin, lung, or disseminated infection (Patterson, 2010; Denning, 2015). The pulmonary tract is the major target system for *Aspergillus* spp. Pulmonary involvement is represented in several clinical entities: the allergic broncho-pulmonary aspergillosis (ABPA), aspergilloma, chronic pulmonary aspergillosis (CPA), and invasive pulmonary aspergillosis (IPA), leading possibly to disseminated invasive aspergillosis (IA). Each of these entities, differing in the symptoms and epidemiological parameters, differ as well in regard to diagnosis and therapy.

Diagnosis of human fungal infections in general, including that of *Aspergillus* spp. infections, is based on demonstration of the etiological agent directly in the clinical specimen by various microbiological or histopathological techniques, followed by culturing the agent from the clinical sample and identifying it (Balaye and Brandt, 2011).

Current laboratory diagnosis of aspergillosis employs also non-cultural methods, such as immunological assays, including detection of anti-*Aspergillus* antibodies and the *Aspergillus* antigen-galactomannan (the Platelia test). Additional non-cultural tests include detection of *Aspergillus* nucleic acids in patients’ blood or other clinical samples, by using PCR technology (Balaye and Brandt, 2011; Denning, 2015).

At the situation of success of the classical diagnostic venue leading to isolation and identification of the etiological agent—the rational approach is attempting to manage the infection with specific therapy. Specific antifungal therapy relies on determination of the sensitivity of the isolated fungal agent to antifungal drugs. Hence, assessment of susceptibility of the identified agent to the available antifungal agents is an essential step of the diagnosis in the process of management of the specific infection, as was pointed out in a recent publication in regard to diagnosis of aspergillosis (Powers-Fletcher and Hanson, 2016).

Therapy of pulmonary aspergillosis is based on the use of voriconazole as a drug of choice. Posaconazole, isavuconazole, liposomal amphotericin B, or amphotericin are used as well. For allergic forms of aspergillosis such as ABPA or allergic *Aspergillus* sinusitis, the recommended treatment is itraconazole. Corticosteroids may also be helpful.

Invasive pulmonary aspergillosis and IA are infections associated with high mortality rates, in many cases even under treatment (Denning, 2015). Thus, combination therapy, with more than a single antifungal drug, particularly with drugs of different modes of activity, seems as a sensible approach that could improve the management of such infections (Spitzer et al., 2017). As a consequence, susceptibility testing of antifungal drug-combinations against fungi evolved as part of the management of aspergillosis.

The following text will concentrate on description of susceptibility assessment of antifungal drugs and antifungal drug-combinations against *Aspergillus* spp.

### Susceptibility Testing of Antifungals—General Overview

Susceptibility of antimicrobial drugs has historically started with the era of antibiotics in use against bacterial infections, both in terms of methodology, evaluation, and breakpoint determination.

Susceptibility of antifungal drugs was introduced into research and clinical use significantly later. The major reason for this delay was the paucity of availability of antifungal drugs in addition to technical difficulties of standardization of such assays for fungi, particularly for molds (Johnson et al., 2011).

The necessity for susceptibility testing to antifungals arouse with the development during the last decades of new antifungal drugs and the emerging problem of appearance of resistant fungal species or fungal strains within a given species. The greater arsenal of antifungals, acting by different modes, led also to use of combinations of antifungals, which in turn led to susceptibility testing of antifungal drug combinations.

Most of the methods for in vitro susceptibility assays are based on the guidelines developed by the Clinical and Laboratory Standards Institute (CLSI) (Clinical and Laboratory Standarts Institute, 2008) in the United States (Johnson et al., 2011) and its European Equivalent—The European Committee on Antimicrobial Susceptibility Testing (EUCAST) (Cuenca-Estrella et al., 2003).

In terms of antifungal susceptibility, the CLSI developed methods for yeasts—the M27-A3 document and M38-A2—for molds, respectively. Both are based on use of the broth dilution method: the macro-dilution and micro-dilution. These methods enable establishment of minimal inhibitory concentrations (MICs) and in some instances breakpoints.

The broth dilution methods can be evaluated visually or by spectrophotometry (Clinical and Laboratory methods). The latter enables in addition to MIC determination, also 50% of MIC, or values in between.

A modification of the broth method which can also assess MIC values is the *E*-test (Espinel-Ingroff, 2001). The *E*-test is an agar-based technique in which a paper strip loaded with a concentration-gradient of an antimicrobial drug is placed on an agar plate seeded with the test microbe, allowing readings of the results by measuring an ellipse zone of inhibition.

An additional agar-based technique is the disk diffusion method which does not determine MIC but measures inhibition zones. The agar-based methods are evaluated visually.

Lee et al. (2009) compared the disk diffusion assay with the *E*-test in susceptibility tests of Candida and found a correlation between the two methods. The study of Salas et al. (2013) explored the predictive values of *in vitro* susceptibility testing of *A. fumigatus*, using both the micro-dilution and disk diffusion assays. These investigators concluded that the susceptibility data correlated with the *in vivo* data in an experimental animal model. An additional study of Lass-Flörl et al. (1998) have shown it in clinical cases of human aspergillosis, in patients with *A. fumigatus, A. flavus*, or *A. terreus* infection.

Reliable antifungal susceptibility testing requires standardization of the procedures, including medium, fungal inoculum, temperature of incubation, time of reading, and result interpretation (Johnson et al., 2011). It also needs adjustment as to the class of antifungals. Thus, establishing MICs of molds, such as *Aspergillus*, spp. to echinocandins is problematic, since no complete macroscopic growth inhibition is observed, but rather partial inhibition is noted, which is associated with abnormal hyphae formation. Hence, a different criterion for susceptibility to echinocandins was introduced: the minimal effective concentration (MEC), which defines the minimal antifungal concentration which causes the morphological changes (Kurtz et al., 1994).

In addition to MICs or MECs, susceptibility testing enables also the determination of the drug’s fungicidal activity, by defining the minimal drug concentration causing fungal death—minimal fungicidal concentration (MFC) (Pfaller et al., 2004).

The advantages and shortcomings of the different susceptibility test methods are summarized in **Table 1**.

**Table 1:** Advantages and shortcomings of antifungal susceptibility testing of *Aspergillus* species.

Antifungal class	Broth dilution methods		Agar based methods	
		
	Macro-broth dilution	Micro-broth dilution	Disk diffusion	E-Test
**Polyenes**				
Advantages	Enable establishment of MIC^∗^; Visual & spectrophotometric evaluation	Enable establishment of MIC; Visual & spectrophotometric evaluation	Simple, fast, affordable	Can assess MIC; Less laborious than micro- broth dilution
Shortcomings	Labor intensive and massive use of reagents and supplies.	Labor intensive, but lesser use of reagents and supplies	No MIC can be established	Difficult to determine end point
**Azoles**				
Advantages	Enable establishment of MIC^∗^; Visual & Spectrophotometric evaluation	Enable establishment of MIC; Visual & spectrophotometric evaluation	Simple, fast, affordable	Can assess MIC; Less laborious than micro-broth dilution
Shortcomings	Enable establishment of MIC; Visual & spectrophotometric evaluation	Labor intensive and massive use of reagents and supplies	No MIC can be established	Maybe difficult to determine the end point
**Echinocandins**		
Advantages	Activity based on fungal morphological changes can be assessed by microscopic evaluation	Activity based on fungal morphological changes can be assessed by microscopic evaluation	Simple, fast, affordable	Less laborious than micro- broth dilution
Shortcomings	Only MEC^∗∗^ can be established and not MIC	Only MEC can be established and not MIC	Zone of inhibition maybe difficult to determine due to possibility of residual growth	Difficult to determine end point due to possibility of residual growth

*^∗^MIC, minimal inhibitory concentration; ^∗∗^MEC, minimal effective concentration.*

### Susceptibility Testing of Antifungals Against *Aspergillus*

Susceptibility testing of *Aspergillus* spp. is an established tool in clinical mycology in institutions dealing with invasive mycoses patients and also a tool for epidemiological studies.

Since triazoles are the major line of antifungal drugs in treatment of IA, many of the susceptibility studies on *Aspergillus* spp. focus on these drugs. An example of such studies is the investigation of Baddley et al. (2009) who assessed patterns of susceptibility of 274 clinical isolates of *Aspergillus* spp. in transplant recipients, using the M38-A2 broth dilution method. They assessed susceptibility to itraconazole, voriconazole, posaconazole, and ravuconazole and also amphotericin B. Interestingly, the authors report that no significant relationships of MIC and mortality were noted. A later study by Pfaller et al. (2011) examined a 9-year susceptibility trend (2001–2009) of *Aspergillus* spp. to triazoles (itraconazole, posaconazole, and voriconazole). These authors concluded that decreased susceptibility among *Aspergillus* spp. was observed; however, there was no consistent trend toward decreased susceptibility for any triazole in *A. fumigatus* or *A. flavus* over time.

In view of the increasing resistance to triazoles and concerns of cross resistance, Gregson et al. (2013) examined the susceptibility of *A. fumigatus* clinical isolates to isavuconazole in comparison to that of the older triazoles: itraconazole, voriconazole, and posaconazole. They found that isavuconazole MICs were higher in strains with reduced susceptibilities to other triazoles.

A recent publication by Sanguinetti and Posteraro (2017) points to the importance of introduction of newer technologies for antifungal susceptibility testing in view of the rising problem of resistance. Such technologies, which are based on detection of specific mutations in fungi, are not yet available in clinical settings for *Aspergillus* spp. susceptibility testing.

As indicated afore, susceptibility testing of molds to echinocandins may be problematic due to inability to establish clear-cut MIC values, but is evaluated by the MEC. A recent study in Brazil (Denardi et al., 2017) explored by the broth micro-dilution method of EUCAST and using the EUCAST-proposed breakpoints the *in vitro* susceptibility of 105 clinical and environmental strains of *A. fumigatus* and *A. flavus* to the antifungal drugs: amphotericin B, azoles, and echinocandins. They found that there were differences among the echinocandins (caspofungin, micafungin, and anidulafungin) as to activity and also variability as to susceptibility of the *Aspergillus* spp.

Susceptibility testing is of special importance regarding infections caused by *A. terreus*, known for its relative resistance. Lass-Florl et al. (2005) studied 67 cases of IA caused by *A. terreus* and non-*A. terreus* (32 vs. 35, respectively) regarding susceptibility to amphotericin B, voriconazole, and caspofungin. *In vitro, A. terreus* was found to be resistant to amphotericin B; the infections were associated with a lower response rate to amphotericin B therapy and had a poor outcome.

A relative recent aspect, consequence of the use of antifungal drugs, is the emergence of resistance in fungi to antifungal drugs, particularly to triazoles. As triazoles are the mayor group of drugs in therapy of aspergillosis the issue of resistance is most relevant in this context. It is believed that the increase in resistant *Aspergillus* strains is associated with the massive use of azole compounds in agriculture (Meis et al., 2016).

### Susceptibility Testing of Antifungal Drug Combinations—General Overview

As indicated earlier, with the increase in number of antifungal drugs of different classes enabling the use of drug combinations, it became necessary to evaluate the efficacy of such combinations *in vitro* and *in vivo* against the fungal pathogens. This led to susceptibility testing of antifungal drug combinations.

Susceptibility testing of antifungal drug combinations aims to determine, whether:

1. The combination may improve the management of the infection over that of each of the drugs in the combination, which would mean synergy.
2. The combination may worsen the management of the infection more than each of the drugs in the combination, meaning antagonism.
3. The combination does not affect the management of the infection vs. the treatment with each of the drugs in the combination, hence indicating indifference.

The preferred method for assessment of drug combinations is the checkerboard technique (Chiou et al., 2001; Semis et al., 2015), that is based on the micro-dilution broth assay.

#### A. Checkerboard Method

This technique determines the MIC of each drug in the combination and the activity of the combination. By using a formula which takes into account the mutual effects of each drug on the other component, it is possible to determine whether there is a synergistic or antagonistic effect by using the two drugs together, or no effect at all compared to the single drug.

A concentration gradient of each drug in the combination is prepared in 96-well microtiter plates in a two-dimensional manner. One hundred milliliters of test fungal suspension at concentration of 1–5 × 10^4^ conidia/ml is added to each well (end result 200 μl/well). The plates are incubated at 37°C for 48°h. The MICs are determined as the lowest drug concentration with no visible growth. For echinocandins the MEC, indicating the lowest concentration causing abnormal hyphal growth, is estimated by microscopy (inverted microscope).

Drug interactions are evaluated by an index—the fractional inhibitory concentration index (FICI), which takes into account the MIC of drug A (MICA) and MIC of drug B (MICB) in the combination and as single drugs. The FICI is obtained by calculating: MICA in combination/MICA alone + MICB in combination/MICB alone. FICI values of up to 0.5 are considered as synergy, 0.5 to <4 indicate indifference and FICIs of 4 or above suggest antagonism (Chiou et al., 2001; Odds, 2003).

#### B. Disk Diffusion Method

Disk diffusion method is an additional recognized technique to assess *in vitro* susceptibility of microorganisms to various drugs (Johnson et al., 2011).

This technique can be adapted also for assessment of drug combinations. Specifically, the test organism is spread on agar plates which contain in the medium one of the drugs in the combination and the second drug is incorporated in the paper-disks placed on the agar plates.

Following incubation (generally 24/48 days at 37°C) the zone of inhibition is measured. To determine synergy, antagonism or indifference the inhibition zone is compared to the inhibition zones around paper disks entailing the individual drugs of the combination placed on agar plates spread with the test fungus. Larger inhibition zones on the combination plate would indicate synergy, smaller inhibition zone antagonisms, and a similar inhibition zone indifference.

### Susceptibility Testing of Antifungal Drug Combinations Against *Aspergillus*

Therapy of the major invasive human fungal infections by antifungal combinational therapy was summarized recently by Spitzer et al. (2017) in a comprehensive, thorough review article.

Antifungal combination therapy is known since some decades in the clinical setting of meningeal cryptococcosis, where combining amphotericin B and 5-fluorocytosin results in the improvement of the outcome (Bennett et al., 1979). This combination therapy is considered as gold standard for treatment of cryptococcal meningitis by WHO guidelines (Perfect and Bicanic, 2015; Maziarz and Perfect, 2016).

In addition, the combination of amphotericin B and fluconazole is efficacious also in treating *Candida meningitis* (Smego et al., 1984).

Combination treatment of IA is thus far considered in high risk patients, but not as a general recommendation (Cadena et al., 2016).

As to other mycoses no specific guidelines for clinical settings are available and most of the published literature includes experimental studies both *in vitro* and *in vivo*.

Sionov et al. (2005) investigated in an experimental *in vivo* model in mice infected with *A. fumigatus* the efficacy of therapy with polyenes in comparison to that of the combination of polyenes and echinocandins. Specifically, the infected mice were treated with:

(1) amphotericin B or with a lipid formulation of amphotericin B: amphotericin B-intralipid;
(2) with a combination of the polyenes (amphotericin B/amphotericin B-intralipid) and the echinocandin, caspofungin.

These experiments showed that mice treated with the combination of the two drugs had higher survival rates, prolonged survival time, and lower fungal visceral colonization.

The authors concluded that a combination of drugs acting by different modes of activity, such as the polyenes, affecting the fungal cytoplasmic membrane and the echinocandins, acting on the fungal cell wall, result in a better outcome.

A number of investigators used a similar approach in experimental infections. A few examples: Santos et al. (2017) reported on the efficacy of fluconazole and amphotericin B in controlling a *Cryptococcus gattii* infection in a murine model of cryptococcosis. The combination treatment revealed improvement in survival and reduced morbidity. Another study (Chen et al., 2013) reported that posaconazole exhibits *in vitro* and *in vivo* synergy with caspofungin against drug susceptible or resistant *C. albicans* strains. The authors indicate the potential therapeutic applicability of such combinations.

As to combinational antifungal therapy in experimental aspergillosis there are also several studies (Petraitis et al., 2009, 2017; Zhang et al., 2014). Petraitis et al. (2009) studied in rabbits the activity of the combination of the echinocandin–anidulafungin and the triazole–voriconazole against IPA. They found a synergetic effect expressed in reduction in several parameters of the pulmonary infection vs. treatment with each of the drugs alone. The same group of investigators reported in a recent publication (Petraitis et al., 2017), that by using the same concept and same model, with a different combination of antifungals, namely the newer triazole—isavuconazole and a different echinocandin—micafungin, similar results were noted. Thus, enforcing the validity of the concept.

Another interesting study by a different group of investigators (Zhang et al., 2014) explored the combination of voriconazole and caspofungin against invasive pulmonary infection in neutropenic rats caused by different *Aspergillus* species: *A. fumigatus, A. flavus*, or *A. niger*. The authors overall conclusions were that the combination had a synergistic effect against infection caused by *A. flavus* and *A. niger*, but only minor improvement in infection caused by *A. fumigatus*.

These studies emphasize the complexity and difficulty to draw clear-cut conclusions as to the concept of combinational antifungal therapy.

As to *in vitro* susceptibility testing of antifungal combinations against *Aspergillus* spp.—here too are a number of reports in the literature. A recent article by Denardi et al. (2017) reports on susceptibility testing of *A. fumigatus* to antifungal–drug combinations. The authors evaluated combinations of triazoles and echinocandins on itraconazole-resistant strains, using two methods: the checkerboard assay and the *E*-test. The data generated, MIC and MEC, at two different time readings (24 and 48 h) were compared. The analysis of the data showed that the correlation coefficient between the methods depended on the reading-time and the specific combination. The combinations of the azoles and echinocandins measured by the *E*-test showed synergy when readings were done after 24 and 48 h, albeit the effect was reduced at the later reading point, at which some combinations revealed indifference. This study points to the difficulty of getting clear-cut conclusions, as they may be influenced by the method.

An additional recent interesting study of Pfaller et al. (2009) reports on the susceptibility of azole-resistant *A. fumigatus* and other molds and yeasts, to the combination of azoles with Hos2 fungal histone deacetylase (HDAC) inhibitor MGCD290. The study showed that the activity of fluconazole plus MGCD290 was synergistic against 6/10 *Aspergillus* isolates.

A different combination of echinocandins with the investigative anti-chitin synthase compound, nikkomycin, was explored against *A. fumigatu*s and other molds (Chiou et al., 2001). The investigators found synergy for *A. fumigatus*, and indifference for other *Aspergillus* species: *A. flavus, A. terreus*, and *A. niger.*

*Aspergillus terreus* causes systemic infections in immunocompromised patients. The infection is difficult to treat, as *A. terreus* can be resistant or less sensitive to amphotericin B (Lass-Florl et al., 2005). Semis et al. (2015) used the checkerboard assay to evaluate *in vitro* different antifungal drug combinations against *A. terreus*. Antifungal activity of combinations of nystatin/nystatin-intralipid with voriconazole, caspofungin, terbinafine, or 5-fluorocytosine was assessed. It was noted that combination of nystatin-intralipid with caspofungin exhibited better antifungal activity than each drug alone and resulted in synergy in three out of six tested strains of *A. terreus*, while this was not noted with nystatin and caspofungin. Nystatin intralipid or nystatin with voriconazole yielded indifferent interactions. Nystatin-intralipid and terbinafine showed a strong antagonism in all six *A. terreus* strains tested. Susceptibility of *A. terreus* strains to the drug-combinations nystatin or nystatin-intralipid and terbinafine or 5-flurocytosine was also tested by the disk diffusion assay. The results were comparable with those obtained by the checkerboard assay.

In summary, susceptibility of *Aspergillus* spp. to antifungals or antifungal combinations should be considered in management of aspergillosis. However, the complexity associated with the interpretation of the data, particularly in regard to antifungal combinations, still necessitates additional investigational efforts. These should involve studies to better define the predictive value of the data obtained in the susceptibility tests. Furthermore, if possible, susceptibility data should be matched with specific mutations in fungi in order to avoid resistant strains, as suggested by Sanguinetti and Posteraro (2017).

This review comprised of a combination of texts, which focus both on animal and human aspergillosis, is unique as such. Furthermore, the part dealing with susceptibility of antifungal drugs and combination of drugs, as part of diagnosis in human aspergillosis, is unique as well. These factors taken together make the manuscript relevant within the scope of the Research Topic: “Diagnostic Approaches for Aspergillus Infections.”

## Author Contributions

DE writing of the veterinary part of the review. ES writing of the human part of the review.

## Conflict of Interest Statement

The authors declare that the research was conducted in the absence of any commercial or financial relationships that could be construed as a potential conflict of interest.
